# Serum immunoreactivity to neurofilament-medium shows high sensitivity and specificity in patients with Behçet disease

**DOI:** 10.1136/rmdopen-2024-005100

**Published:** 2025-05-30

**Authors:** Tayfun Hilmi Akbaba, Buket Donmez-Demir, Ayse Ilksen Colpak, Yeliz Z. Akkaya-Ulum, Gizem Ayan, Sefik Evren Erdener, Sibel Kadayifcilar, Aslı Tuncer, Umut Kalyoncu, Banu Balci-Peynircioglu, Turgay Dalkara

**Affiliations:** 1 Department of Medical Biology, Hacettepe University, Ankara, Turkey; 2 Institute of Neurological Sciences and Psychiatry, Hacettepe University, Ankara, Turkey; 3 Department of Neurology, Hacettepe University, Ankara, Turkey; 4 Department of Internal Medicine, Division of Rheumatology, Hacettepe University, Ankara, Turkey; 5 Department of Ophthalmology, Hacettepe University, Ankara, Turkey; 6 Departments of Neuroscience and, Molecular Biology and Genetics, Bilkent University, Ankara, Turkey

**Keywords:** Autoantibodies, Behcet Syndrome, Sensitivity and Specificity

## Abstract

**Objectives:**

Behçet disease (BD) is a complex vasculitis with both autoimmune and autoinflammatory features. Despite specific clinical features, no laboratory tests are available for the diagnosis of BD. We recently found that BD sera exhibited immunoreactivity against neurofilament medium protein (NF-M). This study aimed to replicate this finding in an independent cohort and to assess the specificity and sensitivity of NF-M immunoreactivity in serum samples obtained from BD, systemic lupus erythematosus (SLE), multiple sclerosis (MS), psoriatic arthritis (PsA) and non-Behçet uveitis (NBU) patients as well as healthy donors.

**Methods:**

Serum samples from 76 patients (33 BD, 16 MS, 15 SLE, 9 PsA and 3 NBU) and 22 healthy donors (totalling 98 sera) were analysed. Mouse brain tissue sections were immunolabelled with the sera and examined using confocal microscopy.

**Results:**

97% (32/33) of BD patient sera exhibited a distinct fine filamentous staining pattern consistent with NF-M protein immunolabelling in axons, while sera from healthy controls and patients with SLE, MS, PsA and NBU showed no similar staining. Conversely, MS patient sera displayed a thick filamentous staining pattern attributed to oligodendrocytes and their myelin-forming processes. SLE patient sera intensely labelled all cell nuclei, conforming to immunoreactivity against nuclear antigens.

**Conclusions:**

These findings reveal the ubiquitous presence of NF-M immunoreactivity, reportedly cross-reacting with bacterial heat shock protein 65, in BD sera. This common and specific immunoreactivity may serve as a valuable tool for diagnosing BD. Additionally, the data confirm the unique potential of connective tissue-poor brain sections for identifying sero-immunoreactivity.

**WHAT IS ALREADY KNOWN ON THIS TOPIC**

- Sera from patients with Behçet disease (BD) exhibit immunoreactivity to neurofilament medium (NF-M), which shares common epitopes with bacterial heat shock protein 65 (HSP-65).

**WHAT THIS STUDY ADDS**

- We evaluated the specificity and sensitivity of NF-M immunoreactivity in an independent cohort of patients and controls using immunofluorescent labelling of mouse brain sections with patient sera.
- NF-M immunoreactivity showed high sensitivity and specificity. 97% of BD patient sera stained with a distinct fine filamentous immunostaining pattern, consistent with NF-M in axons, unlike sera from healthy controls and non-BD patient groups.
- Sera from patients with multiple sclerosis showed thick filamentous immunolabelling associated with oligodendrocytes.
- Sera from patients with systemic lupus erythematosus stained cell nuclei, consistent with immunoreactivity against nuclear antigens.

**HOW THIS STUDY MIGHT AFFECT RESEARCH, PRACTICE OR POLICY**

- NF-M immunoreactivity is highly specific and can be used as a diagnostic tool for BD. Its high sensitivity and cross-reactivity with HSP-65 suggest a possible role in BD pathogenesis.
- The study highlights brain sections’ utility in detecting sero-immunoreactivity in autoimmune disorders.

## Introduction

Behçet disease (BD), first described by Turkish dermatologist Hulusi Behçet, manifests as a triple symptom complex characterised by recurrent genital and oral ulcers, along with uveitis.^1^ Currently recognised as a complex vasculitis, BD exhibits autoimmune and autoinflammatory features, stemming from dysregulation in both innate and adaptive immune systems.^2^ Epidemiological studies have emphasised the relatively high prevalence of BD, particularly in the Old Silk Road region. Turkey demonstrates a significant prevalence ranging from 80 to 420 cases per 100 000 individuals.^3 4^ In other regions, the prevalence varies, with Japan reporting 16 cases per 100 000 people, Korea with 2.6 cases per 100 000 people, China with 4 cases per 100 000 people, Iran with 80 cases per 100 000 people, Iraq with 17 cases per 100 000 people, and Jordan with a substantial prevalence of 660 cases per 100 000 people.^5^ These epidemiological findings highlight the potential role of environmental factors in the pathogenesis of BD.

The diagnosis of BD typically involves clinical findings such as recurrent oral and genital ulcerations, ocular lesions, skin manifestations, joint involvement and vascular and central nervous system (CNS) manifestations. Guidelines such as the International Criteria for Behçet Disease (ICBD) and the International Study Group (ISG) criteria are commonly used for clinical diagnosis.^6^ The absence of a specific biochemical or genetic test poses challenges, and the diagnosis relies solely on clinical findings. Despite this, there is growing interest in investigating the role of autoantibodies in the aetiology of BD and their potential use as diagnostic markers. Notably, kinectin,^7^ alpha-tropomyosin,^8^ PTEN-induced putative kinase 1 (PINK1)^9^ and several endothelial cell antigens were identified in this context.^10^ Although autoantibodies against these antigens have been reported in various BD patient cohorts, their low prevalence as well as presence in other autoimmune disorders have restricted their utility as specific markers for BD.

Our research group recently detected immunoreactivity in the sera of patients with BD to the neurofilament medium protein (NF-M). This protein shares common epitopes with the bacterial heat shock protein 65 from Streptococcus sanguinis, a long-standing player in the aetiology of BD. Our initial study convincingly characterised the presence of humoral NF-M immunoreactivity using combined approaches.^11^ While we observed a higher prevalence and specificity compared with previously reported immunoreactivities, the sensitivity and specificity of NF-M immunoreactivity require validation in an independent cohort with statistically adequate number of subjects to identify true positives and negatives. Moreover, our initial study used fluorescent light microscopy to screen immunolabelling of axon bundles with patient sera on mouse brain sections.^11 12^ This microscopic method may have underestimated the thin filamentous immunopositive labelling for NF-M and could not sufficiently differentiate it from the thick filamentous labelling by multiple sclerosis (MS) sera. This limitation arises from the lower sensitivity and resolution of fluorescent light imaging compared with confocal fluorescent microscopy. To assess the potential of NF-M immunoreactivity in BD, our present study aims to analyse NF-M immunoreactivity in an independent sample pool by using confocal microscopy. This pool will encompass patients with systemic lupus erythematosus (SLE), MS, psoriatic arthritis (PsA), non-Behçet uveitis (NBU) and healthy donors, in addition to patients with BD. This broader approach ensures the sufficient statistical power needed for sensitivity and specificity evaluations and may shed light on the potential role of NF-M immunoreactivity in BD, a common health problem in old Silk Road countries with an unknown aetiology.

## Materials and methods

### Study group

A total of 76 patients were enrolled in the study, comprising 33 with BD, 16 with MS, 15 with SLE, 9 with PsA and 3 with NBU. Additionally, 22 healthy donors were included for comparison. Patients were recruited from the neurology, rheumatology and ophtalmology clinics of Hacettepe University Hospitals. The patients with BD were clinically diagnosed by a rheumatologist with 20 years of experience. Patients were also evaluated for fulfilment of the ISG and ICBD. Briefly, the ISG criteria should include recurrent genital lesions, eye involvement, skin involvement and two of pathergy positivity in addition to recurrent oral aphthae. ICBD criteria are calculated based on scoring. Oral aphthae are worth 2 points, genital ulcers 2 points, ocular findings 2 points, skin findings, neurological findings, vascular involvement and pathergy positivity one point each. Four points and more fulfil the classification criteria.

Relapsing-remitting MS (RRMS) patients were diagnosed using the McDonald’s criteria,^13^ and SLE patients with 2019 European League Against Rheumatism/American College of Rheumatology criteria,^14^ PsA patients with CASPAR criteria,^15^ uveitides patients were classified by anatomic location according to Standardisation of Uveitis Nomenclature criteria.^16^ The healthy donor group consisted of adults of any gender aged 18 and above, who did not meet the classic diagnostic criteria for BD and had no prior history of rheumatologic or neurological diseases.

### Serum isolation, preabsorption and indirect immunofluorescence staining

Blood samples from patient groups and healthy donors were collected in BD Vacutainer Hemogard tubes. The samples were separated by centrifugation at 4000 rpm and +4°C for 10 min, then stored at −80°C until immunofluorescence staining. A previously published protocol was employed for indirect immunofluorescence using mouse brain tissue sections.^11 12^ In brief, preabsorption of patient and control sera was performed with lyophilised guinea pig liver at a 1/60 dilution in phosphate-buffered saline (PBS) to decrease non-specific background staining. All sera samples underwent preabsorption. Coronal mouse brain slices, 20 µm thick, were cut using a freezing cryostat (Leica). These slices were fixed with formol (10% formaldehyde solution in PBS) for 4 min and permeabilised with a 1% w/v CHAPS solution (AppliChem) in PBS for the indirect immunofluorescence experiment. Following this, brain sections were blocked for 60 min at room temperature with 10% normal goat serum (Millipore). Subsequently, they were incubated for 70 min at room temperature with the preabsorbed patient or control sera at a 1/60 dilution (in PBS). Secondary labelling was conducted at room temperature for 60 min using fluorescent-conjugated (FITC) goat anti-human IgG antibody (1/200 dilution, Jackson Immunoresearch). To minimise non-specific background binding, slides were washed three times for 5 min with PBS between each step. Brain slices were mounted with Hoechst-33258 solution (Thermo Fisher Scientific) after immunofluorescent labelling to label nuclei for easier tissue orientation. The resulting slides were analysed using a Leica SP8 confocal microscope. The researcher evaluating the neurofilament staining pattern was blinded to the patients’ diagnoses. To ensure blinding, all samples were anonymised and assigned unique codes by a separate investigator prior to analysis. The coding key was only revealed after all evaluations were completed.

### Label-free in vivo imaging of myelinated axons

A Leica SP8 confocal laser scanning microscope, with an oil immersion objective (40X; NA: 1.3), was used for spectral confocal reflectance imaging of myelin.^17 18^ Imaging was performed sequentially under 488 nm, 552 and 638 nm visible laser excitation for each region of interest. Laser outputs were sent through an acousto-optical tunable filter and an RT 15/85 partially reflective mirror. The reflected light was collected using photomultiplier tubes, with detection wavelengths centred at the laser wavelengths and with a 30 nm wide collection window. Z-stacks of 80×80×10 µm^3^ volumes were scanned with 512×512 pixels in x-y and with a Z-step size of 0.5 µm. Corresponding Hoechst and FITC fluorescence were acquired in addition to the three reflectance channels. For postprocessing, three reflectance images were converted to 32-bit format and then arithmetically added together to combine reflective signals at different wavelengths. To increase the signal-to-noise ratio, maximum intensity projections of these images were subsequently processed with a median filter (1-pixel radius), background subtraction (20-pixel window) and finally with a Gaussian filter (s=1-pixel radius). These steps generated the images presented in the figures. All image processing was performed using FIJI/ImageJ (V.2.14.0/1.54f).

## Results

### Demographic and clinical findings of the study group

Patients’ demographic findings and clinical characteristics are listed in tables1^3^.

**Table 1:** Clinical and immunolabelling characteristics of patients with Behçet disease (BD)

Patient code	Gender	Filamentous immunostaining	ISG/ICBD criteria	Oral aphthae	Genital ulcers	Uveitis	Skin involvements	CNS involvements	Others	Disease duration (year)	Manifestations at blood collection	Treatment (ever)	Treatment (at blood collection)
BD1	M	+	+/+	+	+	–	+	-	-	1	–	Methotrexate, steroid, colchicine	Methotrexate, steroid, colchicine
BD2	F	+	-/-	+	–	–	+	-	JI	2	–	Colchicine	Colchicine
BD3	M	+	+/+	+	+	+	+	-	JI	7	Oral aphthae, arthritis	Interferon alpha, colchicine, steroid	Colchicine
BD4	F	+	+/+	+	+	+	+	-	JI	7	Oral aphthae, papulopustular	Colchicine, depocilin	Colchicine, depocilin
BD5	M	+	+/+	+	+	–	+	P	JI	12	Papulopustular, neurological involvements	Colchicine, interferon alpha, infliximab	Colchicine, infliximab
BD6	F	+	+/+	+	+	+	+	P	JI	5	–	Colchicine, azathioprine, steroid, cyclophosphamide	Cyclophosphamide
BD7	M	+	+/+	+	+	+	+	-	DVT	14	–	Colchicine, infliximab	Colchicine, infliximab
BD8	M	+	+/+	+	+	–	+	-	JI	1	–	Colchicine	–
BD9	F	+	+/+	+	+	+	+	P	DVT, PE	9	Oral aphthae, arthralgia	Colchicine, steroid, infliximab	Infliximab
BD10	M	negative	+/+	+	+	+	+	P	-	8	–	Colchicine, azathioprine, steroid, infliximab	Colchicine, infliximab
BD11	M	+	+/+	+	+	–	+	-	DVT	8	–	Colchicine, steroid, interferon alpha, cyclophosphamide	–
BD12	M	+	+/+	+	+	+	+	-	DVT, JI	7	–	Colchicine, coumadin, infliximab, depocilin	Colchicine, coumadin, infliximab, depocilin
BD13	M	+	+/+	+	+	+	+	P	DVT, GIS	2	–	Colchicine, azathioprine, steroid	Colchicine, azathioprine, steroid
BD14	M	+	+/+	+	+	+	+	-	JI	0	–	Interferon alpha	Interferon alpha
BD15	M	+	-/+	+	–	–	+	-	DVT, JI, PE, CT	2	Arthralgia, DVT, oral aphthae	Colchicine, steroid, cyclophosphamide, interferon alpha	Colchicine, interferon alpha
BD16	F	+	+/+	+	+	+	–	Non-P	JI	15	–	Colchicine, azathioprine	Colchicine, azathioprine
BD17	M	+	-/+	+	–	–	+	-	DVT	2	Oral aphthae	Infliximab, colchicine, coumadin	–
BD18	F	+	+/+	+	+	–	+	-	JI	9	–	Colchicine	Colchicine
BD19	F	+	+/+	+	+	+	+	Non-P	DVT, JI, CT, PE	2	Arthralgia	Colchicine, steroid, coumadin, cyclophosphamide, interferon alpha	Colchicine, steroid
BD20	F	+	+/+	+	+	+	–	P	JI	19	–	Colchicine, azathioprine, interferon alpha, steroid	Interferon alpha
BD21	M	+	+/+	+	+	+	+	Non-P	-	7	–	Colchicine, depocilin	Colchicine, depocilin
BD22	F	+	+/+	+	+	+	+	Non-P	JI	7	Posterior uveitis	Colchicine, azathioprine, coumadin, interferon alpha	Colchicine, azathioprine
BD23	F	+	-/-	+	–	–	–	P	JI	11	Oral aphthae, neurological involvements	Colchicine, interferon alpha	Colchicine, interferon alpha
BD24	F	faint	+/+	+	+	+	+	P	JI	7	–	Colchicine, azathioprine, steroid, cyclophosphamide	Cyclophosphamide
BD25	F	+	+/+	+	+	–	+	-	JI	3	Arthralgia, oral aphthae, papulopustular	Colchicine	Colchicine
BD26	M	+	+/+	+	–	+	+	-	DVT	2	–	Colchicine, azathioprine, depocilin	Colchicine, azathioprine, depocilin
BD27	F	+	+/+	+	+	+	+	-	JI, GIS, DVT	6	Papulopustular, GI involvements	Colchicine, azathioprine, azathioprine, interferon alpha	Colchicine, interferon alpha
BD28	M	+	-/-	+	+	–	–	P, Non-P	-	16	Neurological involvements	Colchicine, interferon alpha	Colchicine, interferon alpha
BD29	F	faint	+/+	+	+	+	+	-	JI	4	Papulopustular	Colchicine, steroid, depocilin	Colchicine, depocilin
BD30	F	+	+/+	+	+	–	+	-	JI	1	Oral aphthae	Colchicine	–
BD31	M	+	-/+	+	+	–	–	-	DVT	7	–	Colchicine, azathioprine, coumadin, depocilin	–
BD32	M	+	+/+	+	+	–	+	-	JI	3	Oral aphthae, papulopustular	Colchicine, azathioprine, steroid	Colchicine, azathioprine, steroid
BD33	M	+	+/+	+	+	+	+	-	JI	11	–	Azathioprine, interferon alpha	Azathioprine

A, articular; CNS, central nervous system; CT, cardiac thrombus; DVT, deep venous thrombosis; GIS, gastrointestinal system involvement; ICBD, international criteria for BD; ISG, International Study Group; JI, joint involvement; P, parenchymal; PE, pulmonary embolus.

**Table 2:** Clinical and immunolabelling characteristics of patients with relapsing-remitting multiple sclerosis (RRMS)

Patient code	Gender	Thick filamentous immunostaining (myelin)	Somatic immunostaining (Oligodendrocyte)	CNS areas affected
MS-1	F	+	-	On, BS, Sc, Pv, Cb
MS-2	F	+	+	Sc, Pv, Cb
MS-3	F	+	+	Sc, Pv
MS-4	F	+	-	On, Sc, Pv, Cb
MS-5	F	+	+	Sc, Pv, Cb
MS-6	M	+	+	On, Sc, Pv, Cb
MS-7	M	+	+	Pv
MS-8	F	-	-	Sc, Pv
MS-9	F	+	+	BS, Sc, Pv
MS-10	M	+	+	Pv
MS-11	F	+	-	BS,Sc
MS-12	M	-	-	Sc, Pv
MS-13	F	-	-	Sc, Pv
MS-14	F	+	+	BS, Pv
MS-15	F	+	+	Sc, Pv
MS-16	F	+	+	Sc, Pv

BS, brainstem; Cb, cerebellum; CNS, central nervous system; On, optic nerve; Pv, periventricular; Sc, spinal Cord.

**Table 3:** Clinical and immunolabelling characteristics of patients with systemic lupus erythematosus (SLE)

Patient code	Gender	Nuclear immunostaining	Filamentous immunostaining	Mucocutaneous involvement	Renal involvement	Musculoskeletal involvement	Neurological involvement	ANA seropositivity	Subtype
SLE-1	F	+	–	-	+	+	+	+	ND
SLE-2	F	+	–	-	-	-	+	+	AC-4, AC-5
SLE-3	F	+	–	-	-	+	-	+	AC-4, AC-5
SLE-4	F	+	–	-	+	+	-	+	ND
SLE-5	M	+	–	+	+	+	+	+	AC-4, AC-5
SLE-6	F	+	–	+	-	+	-	+	AC-4, AC-5
SLE-7	F	+	–	+	+	-	-	+	AC-4, AC-5
SLE-8	F	+	–	+	-	-	-	+	AC-1, AC-4, AC-5, AC-19
SLE-9	M	+	–	-	+	-	-	+	AC-4, AC-5
SLE-10	F	+	–	+	-	+	-	+	AC-1, AC-4, AC-5, AC-19
SLE-11	F	+	–	+	-	+	-	+	AC-4, AC-5
SLE-12	F	+	–	+	-	+	-	+	ND
SLE-13	F	+	–	-	-	+	-	+	AC-1, AC-19
SLE-14	F	+	–	-	+	+	-	+	AC-1, AC-4, AC-5
SLE-15	M	+	–	-	-	-	+	NA	AC-1, AC-4, AC-5

AC-1, nuclear homogeneous; AC-4, nuclear fine speckled; AC-5, nuclear large/coarse speckled; AC-19, cytoplasmic dense fine speckled; NA, not applicable; ND, not determine.

Briefly, 33 patients with BD (18 male; 15 female) were aged between 20 and 58 (median=37). They all had oral aphthous lesions, 28 (84.8%) had genital ulcers, 28 (84.8%) skin manifestations, 20 (60.6%) uveitis, 20 (60.6%) joint involvement and 13 (39.4%) neurological involvements, (9 parenchymal, 4 non-parenchymal, 1 both parenchymal and non-parenchymal), 11 (33.3%) vascular involvement (deep venous thrombosis (DVT) in 11, pulmonary thrombus in 3, cardiac thrombus in 2), 2 (6.0%) gastrointestinal system (GIS) involvement. 6/33 (18.2%) of the patients did not meet the criteria for ISG. Two patients (6.0%) did not meet all ISG or ICBD criteria, but sera from both patients with probable BD exhibited neurofilament staining on brain sections. Specifically, the active manifestations observed in patients with BD at the time of sampling included oral ulcers (n=9), ocular (n=1) and skin involvements (n=6), musculoskeletal complications (n=5), neurological symptoms (n=3) and gastrointestinal manifestations (n=1). 17 out of 33 patients were in an inactive state.

MS patients (12 female and 4 male) were aged between 20 and 51 (median=34) and all had RRMS. SLE patients (12 female and 3 male) were aged between 20 and 68 (median=40). Seven had mucocutaneous, 6 renal, 10 musculoskeletal and 4 neurological involvements. 13 of the 15 SLE patients had an ANA titre equal to or higher than 1/320. ANA titre for one patient was not available, whereas another patient who had been followed with SLE diagnosis for several years had an ANA titre of 1/100 despite overt SLE symptoms. Of the 16 MS patients, 10 had ANA titres recorded, which were negative in 3 and varied between 1/100 and 320 in 7 others. Nine PsA patients (5 males) were aged between 27 and 59 (median=46). All patients were active, 7/9 (77.8%) were newly diagnosed PsA patients. The sites of involvement at the time of blood collection were peripheral arthritis in 8 (88.9%), psoriasis in 7 (77.8%), enthesitis in 4 (44.4%), nail in 4 (44.4%), dactylitis in 2 (22.2%) and axial active involvement in 1 (11.1%). All three NBU patients were female, aged 51, 54 and 61. Two patients, diagnosed with ankylosing spondylitis, presented with anterior uveitis, while the third patient had multifocal choroiditis with concurrent optic nerve involvement. One of the ankylosing spondylitis patients receiving anti-TNF alpha treatment exhibited elevated plasma ANA and dsDNA levels. Notably, none of the patients had oral or genital ulcers or any other systemic involvement.

### The staining pattern of immunoreactive sera of patients and controls

**Figure 1:**
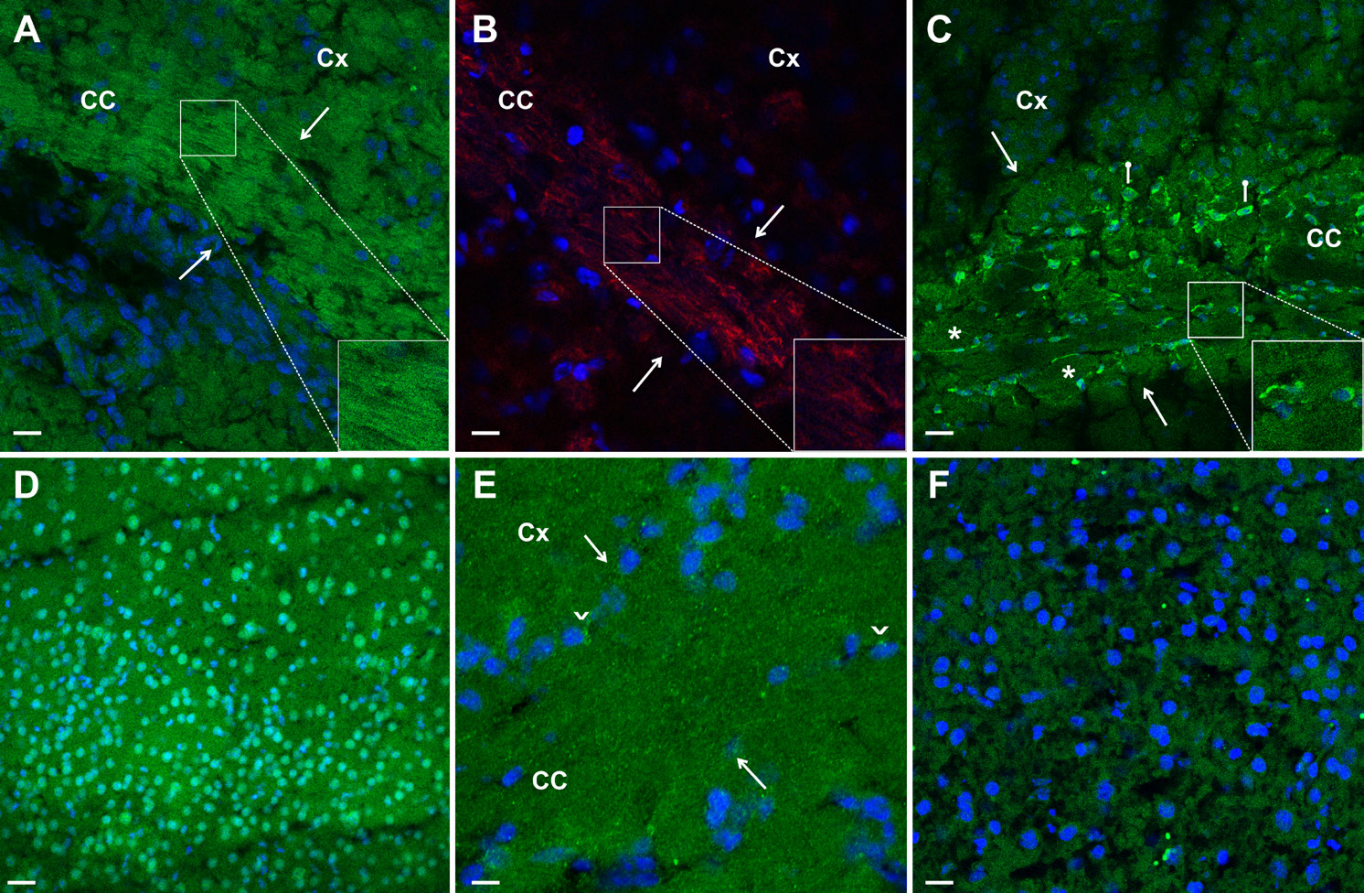
Disease-specific labelling patterns of sera from patients with Behçet disease (BD), multiple sclerosis (MS) and systemic lupus erythematosus (SLE). (**A**) Fine brushstroke-like filamentous staining is evident in the corpus callosum (CC, between white arrows) of the brain section treated with serum from a BD patient. Green depicts FITC-labelled secondary antibody binding against human IgG, while blue marks cell nuclei labelled with Hoechst. (**B**) Staining in the CC (between white arrows) obtained by labelling mouse brain section with a commercial anti-NF-M antibody (red) instead of patient serum is consistent with the filamentous (green) appearance in image A. (**C**) Thicker filamentous labelling (asterisk) and somatic staining (possibly oligodendrocytes, white pin sign) are observed in the CC region (between white arrows) of the brain section treated with a serum from an MS patient. (**D**) The widespread nuclear staining pattern observed in the mouse cortex is the result of indirect immunofluorescence staining performed with the serum of an adult SLE patient. (**E, F**) Negative filamentous staining in brain sections treated with sera from a patient with PsA (**E**) and a healthy control (**F**). PsA patient serum additionally exhibits perinuclear staining in occasional cells (arrowheads). Borders of the CC are marked with white arrows. Cx, cortex; NF-M, neurofilament medium; PsA, psoriatic arthritis.

Immunolabelling of coronal brain sections with BD sera revealed a distinctive pattern characterised by fine brush stroke-like filamentous staining, resembling NF-M protein immunolabelling, as previously reported^11^ (figure 1A,B). Notably, sera from 32 out of 33 patients with BD (97%) displayed this pattern, marking the consistent presence of NF-M immunoreactivity in the sera of patients with BD. Among these 32 patients, 30 exhibited robust staining, while only 2 showed faint filamentous labelling. Conversely, none of the 16 MS or 15 SLE or 9 PsA patients (figure 1E), or 22 healthy controls (figure 1F) exhibited this filamentous staining pattern, indicating that NF-M immunoreactivity assessed with indirect immunolabelling of mouse brain sections may serve as both a sensitive and specific marker for BD. An interim analysis at this stage showed a sufficient statistical power for sensitivity (96.97%) and specificity (100%). Therefore, patient recruitment was halted based on the advice of the monitoring statistician.

**Figure 2:**
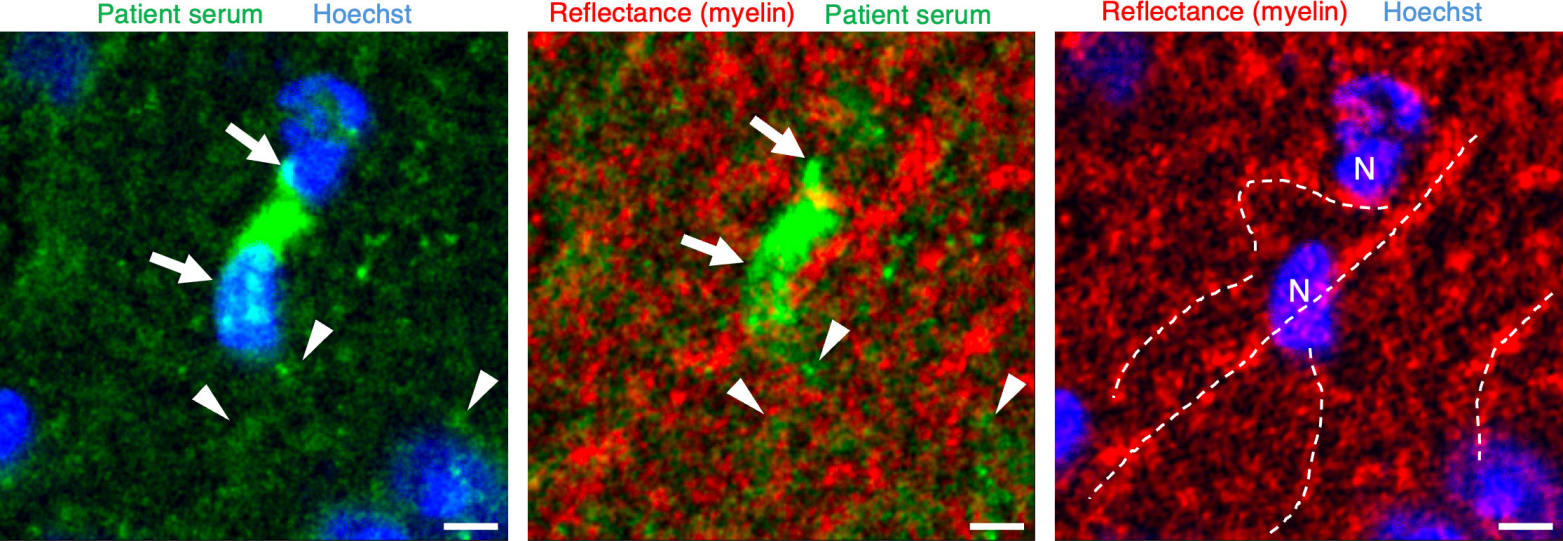
Thick filamentous immunolabelling of mouse brain sections with MS patient serum corresponds to lipid-rich myelin. Arrows indicate the oligodendrocyte soma, arrowheads show extensions immunolabelled relatively fainter with patient serum (green), dashed lines mark myelin tracts and N indicates oligodendrocyte nuclei identified with Hoechst-33258. Scale bar: 5 µm. MS, multiple sclerosis.

In contrast, sera from 13 of 16 MS patients (87%) exhibited a thick and short filamentous staining pattern, often adjacent to or encompassing a cell soma resembling oligodendrocytes, with identifiable processes emanating from the soma (figure 1C). To validate whether these cell somas were indeed oligodendrocytes, we combined fluorescence imaging (serum immunopositivity) with reflectance imaging, which can identify lipid-rich structures like myelinated axons without necessitating immunolabelling because immunolabelling was confounded by compatibility issues between patient serum and secondary antibodies^17^ (figure 2). We hypothesise that the labelling of myelin in MS results in a thicker appearance compared with NF-M labelling within the axon. This is likely because myelin is known to significantly increase the visible axon diameter by more than two times.^19 20^

In SLE patients, no filamentous labelling but widespread distinct nuclear staining consistent with the presence of antinuclear immunoreactivity was observed in all patients (figure 1D) and one healthy donor. None of the 15 SLE sera exhibited the fine or thick filamentous labelling patterns observed in BD or MS patients. Despite mildly positive ANA titres in seven patients with MS, none displayed nuclear staining seen in patients with SLE. Because the sporadic somatic labelling in MS included the perinuclear cytoplasm, it could be easily distinguished from the solely nuclear labelling seen in SLE. Whether this discrepancy is related to a threshold ANA concentration required for nuclear immunolabelling with our protocol or to the presence of immunoreactivity to different nuclear proteins in SLE and MS remains a subject for future investigation.

**Figure 3:**
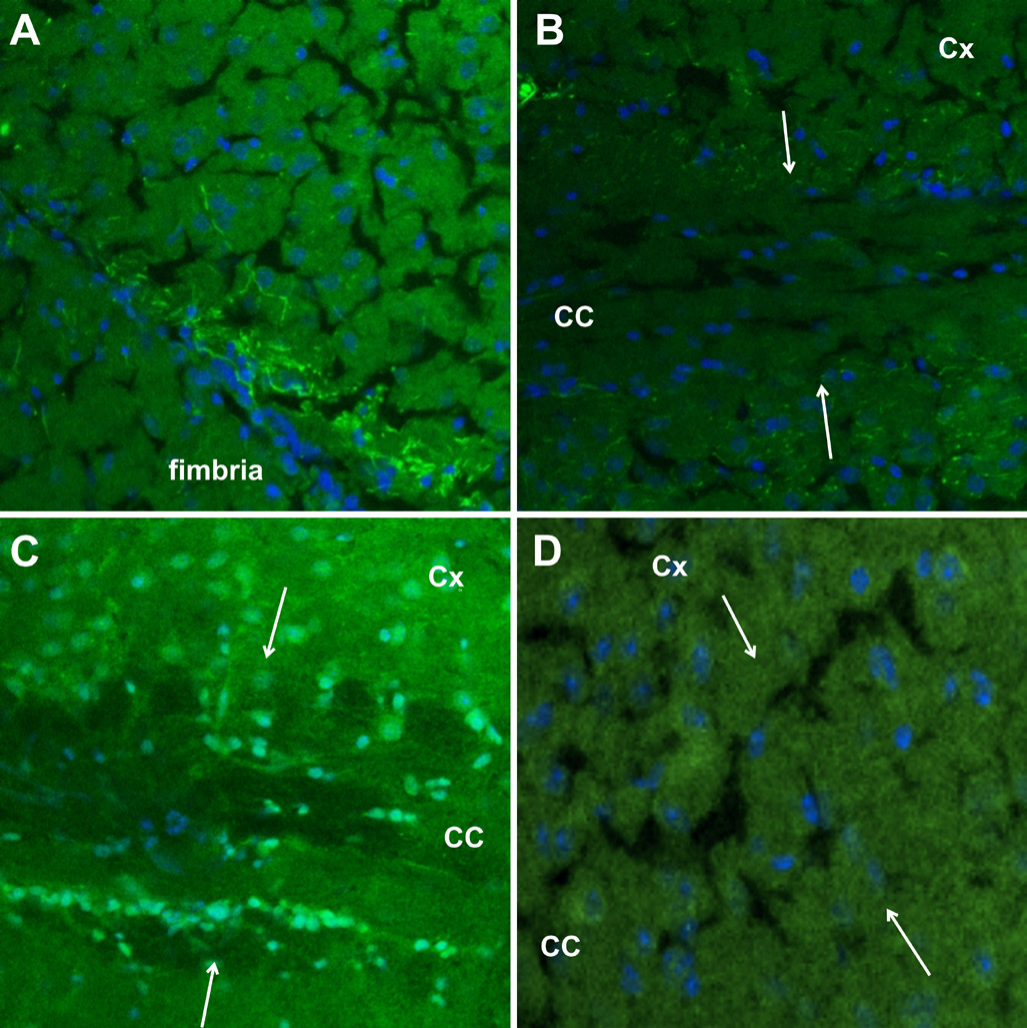
Indirect immunofluorescence staining of mouse brain tissue sections with non-Behçet uveitis patient sera. (**A**) Image taken from the fimbria region of a serum-treated brain section of a patient with bilateral uveitis, showing possible capillary labelling. (**B**) A similar but less intense staining pattern is observed in the corpus callosum (CC)-cortex junction in the same section. (**C**) Diffuse nuclear staining is seen with serum of non-Behçet uveitis patient with elevated plasma ANA and dsDNA levels, resembling the pattern observed in SLE. (**D**) Cellular labelling with serum of another non-Behçet uveitis patient at the CC-cortex junction. Cell nuclei are labelled with Hoechst. Borders of CC are marked with white arrows. SLE, systemic lupus erythematosus.

No filamentous labelling was observed with NBU sera. However, consistent with the heterogeneous nature of patients with different types of uveitis, each of the three patients examined exhibited different immunostaining patterns. Sera from one patient with bilateral uveitis produced a pattern suggesting labelling of branching capillaries (figure 3), while another patient having ankylosing spondylitis and elevated plasma ANA and dsDNA levels possibly secondary to anti-TNF alpha treatment showed widespread nuclear staining similar to that observed with SLE sera. The third patient’s sera caused faint cytoplasmic staining in cell somas.

## Discussion

Our results show the widespread presence of NF-M immunoreactivity in the sera of patients with BD, demonstrating high sensitivity across the majority of cases. Notably, this NF-M immunoreactivity was absent in sera from individuals with MS, SLE, NBU and PsA. This emphasises the specificity of NF-M immunoreactivity for BD, holding promise for its potential application as a laboratory test in the diagnosis of BD, particularly in clinically applicable immunoassay formats. Additionally, the observed sensitivity prompts further research into its potential etiological role, considering NF-M’s molecular mimicry with bacterial heat shock protein 65 (HSP-65). HSP-65, derived from oral Streptococcus sanguinis, has significant homology with human HSP-60.^21^ This high level of homology suggests cross-reactivity between human and bacterial HSP, potentially inducing the proliferation of autoreactive T cells, which may contribute to the immune dysregulation observed in BD.^11^

The diagnosis of BD is typically based on clinical findings assessed by clinicians, supplemented by two different international classification criteria for guidance. However, in everyday clinical practice, there exists a subset of patients who may not pass the threshold of these criteria despite clinicians strongly suspecting BD. This group may exhibit clinical features such as characteristic uveitis changes, CNS involvement like brainstem manifestations, thrombotic events (DVT/pulmonary embolus/cardiac thrombosis) in young individuals, specific patterns of arthritis, and GIS involvement, which are often considered diagnostic clues by experienced clinicians but are not explicitly covered in the ISG/ICBD criteria. In our study, we observed that some patients who presented with relatively severe manifestations of BD but did not satisfy the ISG criteria showed positive immunostaining that we hypothesised to be related to BD. This supports the idea that serum immunoreactivity to NF-M could serve as a diagnostic marker, particularly in challenging cases. This approach highlights the clinical complexity of diagnosing BD and underscores the potential utility of laboratory tools beyond strict adherence to classification criteria, particularly in cases where the clinical picture strongly suggests BD despite not meeting all formal criteria.

The spectrum of immunologic diseases ranges from autoinflammatory to autoimmune disorders.^22^ SLE belongs to the classical polygenic autoimmune disease group, while MS is widely regarded as an autoimmune disorder. In both, increased concentrations of neurofilament-light (NF-L) in CSF serve as a marker of neuronal injury.^23^ When present, antibodies to NF-L could potentially mimic anti-NF-M immunolabelling of brain sections with BD serum.^24^ However, we did not observe the fine filamentous immunolabelling characteristic of BD sera under these conditions. BD displays a mixed autoinflammatory to autoimmune pattern, as does PsA, making PsA a suitable disease control group for comparison with BD.^22^ Therefore, PsA was selected and studied as a disease control group. However, like SLE and MS, PsA sera also did not display a filamentous staining, nor did it exhibit any specific immunostaining pattern. In contrast, NBU, which can sometimes be challenging to differentiate from BD, did not also exhibit the filamentous staining but displayed variable immunolabelling patterns.

We were able to consistently distinguish the characteristic brush-stroke-like labelling pattern of BD sera from non-specific staining of blood vessels or cell bodies, if any. The non-specific labelling never caused BD-like labelling and was relatively rare owing to preabsorption of sera. This fine filamentous labelling pattern in BD was notably different from the thick filamentous staining pattern observed in MS and the sharp nuclear staining seen in SLE. Moreover, additional labelling of cell bodies of oligodendrocytes, along with myelin processes originating from the soma, further facilitated the differentiation of filamentous labelling between MS and BD. Indirect immunostaining of brain sections lacking connective tissue proved to be an effective screening tool for detecting NF-M immunoreactivity, distinguishing it from immunolabelling patterns associated with diseases that pose a challenge in the diagnosis of BD. The nuclear labelling observed in SLE, known to be against nuclear proteins forming the basis of the LE cell test, was differentiated from BD fine immunolabelling, which is caused by autoantibodies against the NF-M protein in neurons. This was reconfirmed by demonstrating parallel labelling between BD serum and a commercially available anti-NF-M antibody, confirming our previous study.^11^ Both approaches labelled neuronal processes, particularly axon bundles in structures like the corpus callosum and fornix. We also verified that the labelling by MS sera reflects an epitope in myelin-forming oligodendrocytes, showing colocalisation with serum immunolabelling and high intensity myelin reflectance sharply delineated from surrounding tissue. While several myelin epitopes have been reported in MS, we did not investigate the specific epitope/s correlating with the thick filamentous immunolabelling in the present study.

Our findings have surpassed initial expectations, revealing a higher-than-anticipated positive NF-M immunoreactivity in patients with BD. The use of confocal microscopy played a crucial role in this detection, enabling the identification of faint labelling with certain BD sera and allowing differentiation between fine and thick filamentous labelling. The sensitivity and specificity observed with the indirect immunofluoresence method also far exceeded the results obtained through western blotting, as reported in our previous study.^11^ We attribute this distinct separation of immunolabelling with BD sera from patient and healthy control sera to the in situ labelling of the NF-M epitope on the structurally intact neurofilaments, allowing for specific binding compared with the methods targeting denatured proteins such as Western blotting or ELISA. These promising results suggest that a more diverse cohort, including patients with varying disease severities and treatment histories, could improve sensitivity analyses in future studies. Additionally, including diseases with overlapping clinical manifestations—such as Crohn’s disease, aphthous stomatitis or retinal vasculitis of other aetiologies—would strengthen the specificity analysis. The current findings support the potential use of NF-M in the diagnosis of BD, highlighting its clinical utility. Further exploration of the role of NF-M in the pathophysiology of the disease is warranted based on these encouraging findings.

Although indirect immunofluorescence labelling has demonstrated high sensitivity and specificity, its practicality is hindered by the time-consuming nature of evaluation and the requirement for an experienced expert. We acknowledge the challenge of non-specific intense staining, particularly for inexperienced personnel, as observed in non-brain tissue samples based on our previous experience. The limited presence of connective tissue in brain tissue sections, unlike peripheral tissues and cell lines expressing a broader range of proteins, reduces but does not eliminate this challenge. While we have established the utility of NF-M-based indirect immunofluorescence labelling for the diagnosis of BD, we recognise the necessity for new approaches beyond this method given their potential clinical applicability. Additionally, we propose further investigation into the potential role of NF-M in the pathophysiology of BD through advanced studies.

## Data Availability

Data are available on reasonable request.
